# Supplementary material for: Anti-Inflammatory and Antifibrotic Potential of Longidaze in Bleomycin-Induced Pulmonary Fibrosis
Source: Life (Basel). 2023 Sep 19;13(9):1932. doi: 10.3390/life13091932 (PMC10532531; doi:10.3390/life13091932)
Supplement: Supplementary file 1 [file life-13-01932-s001.zip › life-2538054-supplementary.pdf]

Table S1. Study Design and Experimental Groups.

Groups	Introduction of bleomycin (day)	Estimation of body weight of animals (day)	Drugs	Euthanasia and sampling
1 – intact control (Intact)	-	d0<br>d7<br>d14<br>d21	-	d7<br>d21
2 – pulmonary fibrosis + NaCl (Vehicle i.m.*)	d0	d0<br>d7<br>d14<br>d21	NaCl<br>intramuscularly	d7<br>d21
3 –pulmonary fibrosis + NaCl (Vehicle i.n.*)	d0	d0<br>d7<br>d14<br>d21	NaCl<br>intranasally	d7<br>d21
4 – pulmonary fibrosis + Pirfenidone	d0	d0<br>d7<br>d14<br>d21	Pirfenidone at a dose 300 mg/kg p.o.*	d7<br>d21
5 – pulmonary fibrosis + LG 60 U/kg i.m.	d0	d0<br>d7<br>d14<br>d21	Longidaze intramuscularly<br>at a dose of 60 U/kg	d7<br>d21
6 – pulmonary fibrosis + LG 120 U/kg i.m.	d0	d0<br>d7<br>d14<br>d21	Longidaze intramuscularly<br>at a dose of 120 U/kg	d7<br>d21
7 – pulmonary fibrosis + LG 1200 U/kg i.m.	d0	d0<br>d7<br>d14<br>d21	Longidaze intramuscularly<br>at a dose of 1200 U/kg	d7<br>d21
8 – pulmonary fibrosis + LG 10 U/kg i.n.	d0	d0<br>d7<br>d14<br>d21	Longidaze<br>intranasally<br>at a dose of 10 U/kg	d7<br>d21
9 – pulmonary fibrosis + LG 30 U/kg i.n.	d0	d0<br>d7<br>d14<br>d21	Longidaze<br>intranasally<br>at a dose of 30 U/kg	d7<br>d21
10 – pulmonary fibrosis + LG 120 U/kg i.n.	d0	d0<br>d7<br>d14<br>d21	Longidaze intranasally<br>at a dose of 120 U/kg	d7<br>d21

* i.m. – intramuscularly; i.n. – intranasally; p.o. – per os.

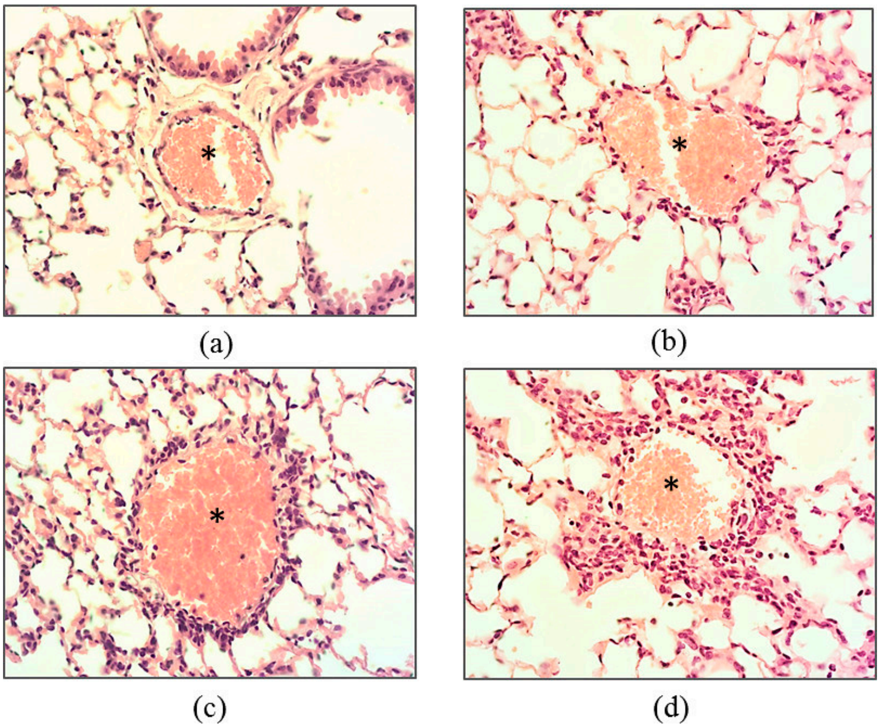

Figure S1. Histology analyses of H&E stained sections: Lung sections graded as (**a**) normal, (**b**) Grade I, (**c**) Grade II and (**d**) Grade III. * Lumen of an artery. Original magnification: ×20

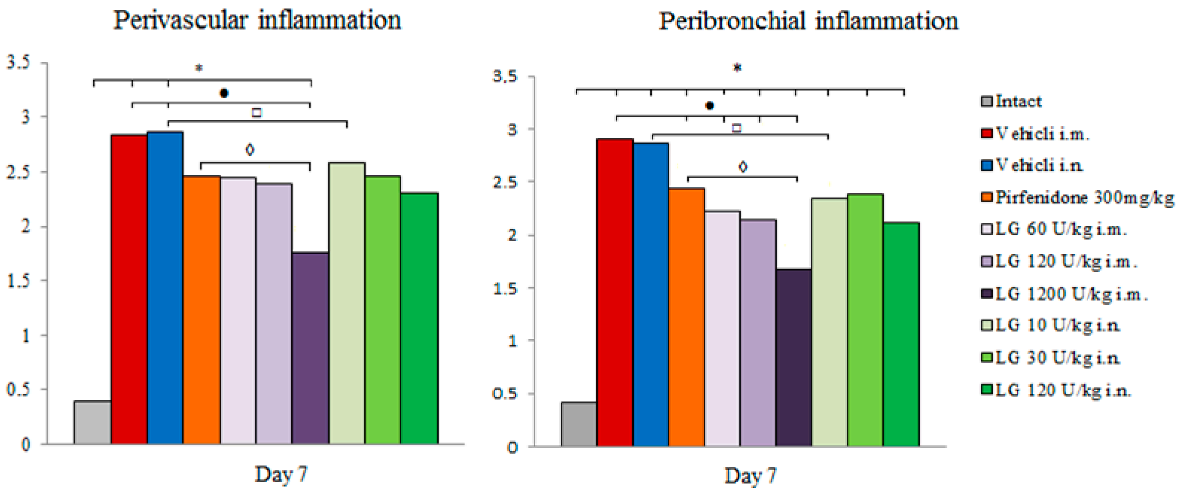

Figure S2. Effect of Longidaze on perivascular and peribronchial inflammation in the lungs of C57BL/6 mice on d7. Groups: Intact - mice of intact control (group 1); Vehicle i.m. and Vehicle i.n. - mice treated with bleomycin (groups 2 and 3); Pirfenidone 300 mg/kg p.o. - pulmonary fibrosis + pirfenidone (group 4); LG 60 U/kg i. m. - pulmonary fibrosis + Longidaze intramuscularly at a dose of 60 U/kg (group 5); LG 120 U/kg i.m. - pulmonary fibrosis + Longidaze intramuscularly at a dose of 120 U/kg (group 6); LG 1200 U/kg i. m. - pulmonary fibrosis + Longidaze intramuscularly at a dose of 1200 U/kg (group 7); LG 10 U/kg i.n. - pulmonary fibrosis + Longidaze intranasally at a dose of 10 U/kg (group 8); LG 30 U/kg i. n. - pulmonary fibrosis + Longidaze intranasal at a dose of 30 U/kg (group 9); LG 120 U/kg i.n. - pulmonary fibrosis + Longidaze intranasal at a dose of 120 U/kg (group 10). P.o. - per os, i.m. - intramuscular, i.n. - intranasal. * $p<0.05$ significance of difference compared with the Intact (Mann–Whitney test); ● $p<0.05$ significance of difference compared with the Vehicle i.m. (negative control for i.m. groups) (Mann–Whitney test); □ $p<0.05$ significance of difference compared with the Vehicle i.n. (negative control for inhalation groups) (Mann–

Whitney test); ◊ p<0.05 significance of difference compared with the Pirfenidone (Mann–Whitney test).

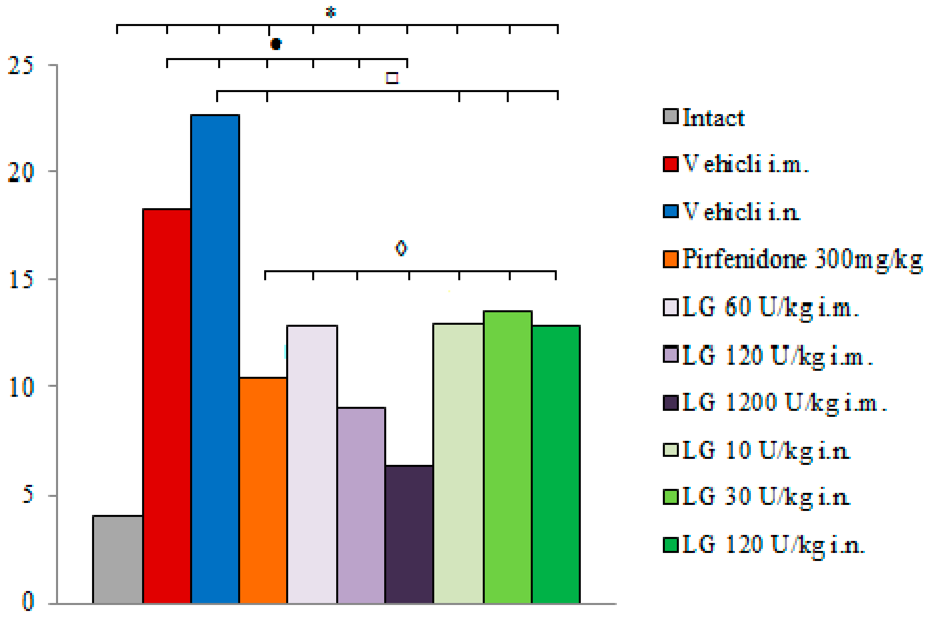

Figure S3. Content of the connective tissue in the lungs of C57BL/6 mice on d21. Groups: Intact - mice of intact control (group 1); Vehicle i.m. and Vehicle i.n. - mice treated with bleomycin (groups 2 and 3); Pirfenidone 300 mg/kg p.o. - pulmonary fibrosis+ pirfenidone (group 4); LG 60 U/kg i. m. - pulmonary fibrosis + Longidaze intramuscularly at a dose of 60 U/kg (group 5); LG 120 U/kg i.m. - pulmonary fibrosis + Longidaze intramuscularly at a dose of 120 U/kg (group 6); LG 1200 U/kg i. m. - pulmonary fibrosis + Longidaze intramuscularly at a dose of 1200 U/kg (group 7); LG 10 U/kg i.n. - pulmonary fibrosis + Longidaze intranasally at a dose of 10 U/kg (group 8); LG 30 U/kg i. n. - pulmonary fibrosis + Longidaze intranasal at a dose of 30 U/kg (group 9); LG 120 U/kg i.n. - pulmonary fibrosis + Longidaze intranasal at a dose of 120 U/kg (group 10). P.o. - per os, i.m. - intramuscular, i.n. - intranasal.

* p<0.05 significance of difference compared with the Intact (Mann–Whitney test); • p<0.05 significance of difference compared with the Vehicle i.m. (negative control for i.m. groups) (Mann–Whitney test); □ p<0.05 significance of difference compared with the Vehicle i.n. (negative control for inhalation groups) (Mann–Whitney test); ◊ p<0.05 significance of difference compared with the Pirfenidone (Mann–Whitney test).

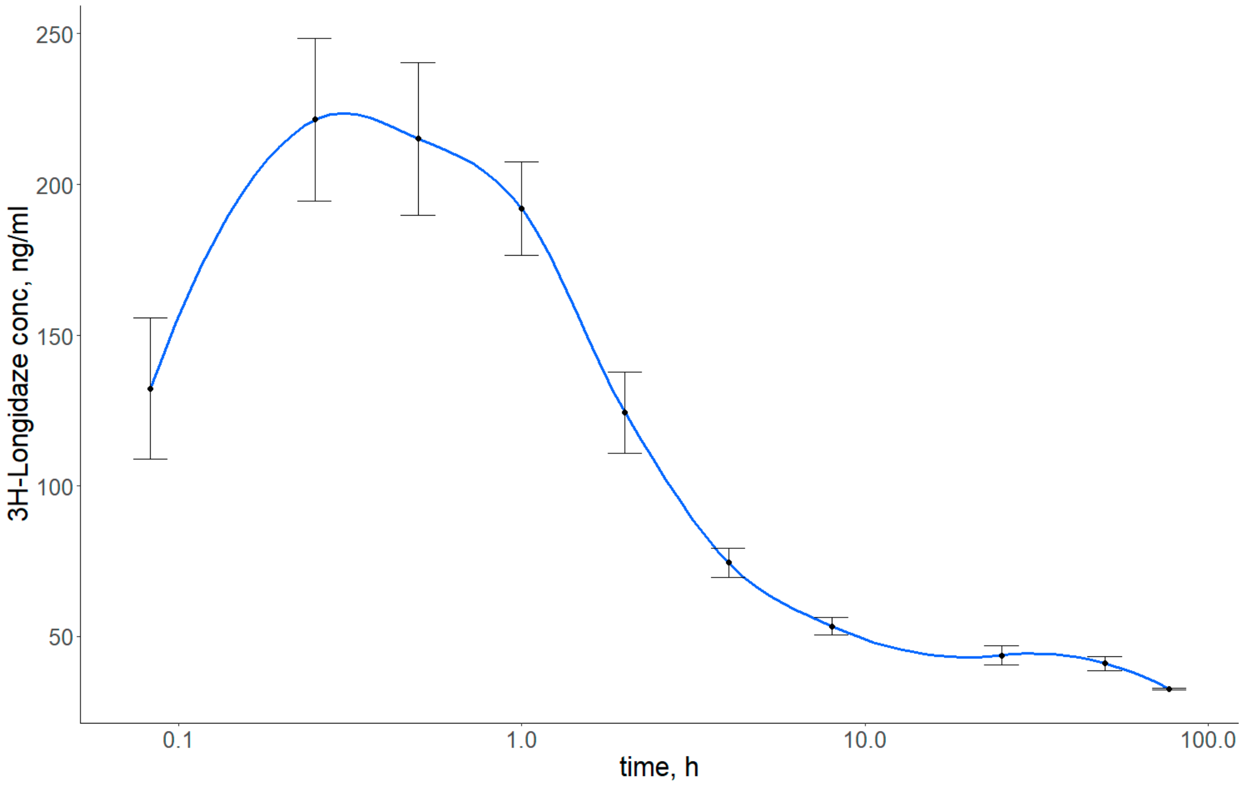

Figure S4. Graph of the Longidaze concentration (ng/ml) versus time after intramuscular injection.
